# Seven-Year Evolution of β-Lactam Resistance Phenotypes in *Escherichia coli* Isolated from Young Diarrheic and Septicaemic Calves in Belgium

**DOI:** 10.3390/vetsci9020045

**Published:** 2022-01-26

**Authors:** Virginie Guérin, Alban Farchi, Damien Thiry, Frédéric Cawez, Paola Sandra Mercuri, Moreno Galleni, Jacques Mainil, Marc Saulmont

**Affiliations:** 1 Bacteriology, Department of Infectious and Parasitic Diseases, FARAH and Faculty of Veterinary Medicine, Uliège, 4000 Liège, Belgium; damien.thiry@uliege.be (D.T.); jg.mainil@uliege.be (J.M.); 2 CEREA, École des Ponts and EDF R&D, IPSL, Île-de-France, 77455 Champs-sur-Marne, France; alban.farchi@enpc.fr; 3 Biological Macromolecules, Center for Protein Engineering (CIP), InBioS, ULiège, 4000 Liège, Belgium; Frederic.Cawez@uliege.be (F.C.); pmercuri@uliege.be (P.S.M.); mgalleni@uliege.be (M.G.); 4 Regional Animal Health and Identification Association (ARSIA), 5590 Ciney, Belgium; marc.saulmont@arsia.be

**Keywords:** β-lactam resistance, calves, evolution of resistance, *E. coli*

## Abstract

Antimicrobial resistance is a major worldwide hazard. Therefore, the World Health Organization has proposed a classification of antimicrobials with respect to their importance for human medicine and advised some restriction of their use in veterinary medicine. In Belgium, this regulation has been implemented by a Royal Decree (RD) in 2016, which prohibits carbapenem use and enforces strict restrictions on the use of third- and fourth-generation cephalosporins (3 GC and 4 GC) for food-producing animals. Acquired resistance to β-lactam antibiotics is most frequently mediated by the production of β-lactamases in Gram-negative bacteria. This study follows the resistance to β-lactam antibiotics in *Escherichia coli* isolated from young diarrheic or septicaemic calves in Belgium over seven calving seasons in order to measure the impact of the RD. Phenotypic resistance to eight β-lactams was assessed by disk diffusion assay and isolates were assigned to four resistance profiles: narrow-spectrum β-lactamases (NSBL); extended-spectrum β-lactamases (ESBL); cephalosporinases (AmpC); and cephalosporinase-like, NSBL with cefoxitin resistance (AmpC-like). No carbapenemase-mediated resistance was detected. Different resistance rates were observed for each profile over the calving seasons. Following the RD, the number of susceptibility tests has increased, the resistance rate to 3 GC/4 GC has markedly decreased, while the observed resistance profiles have changed, with an increase in NSBL profiles in particular.

## 1. Introduction

In Gram-negative bacteria, acquired resistance to β-lactam antibiotics is most frequently mediated by the production of plasmid-encoded β-lactamase (BLA) enzymes hydrolysing the β-lactam ring [1]. There are five categories depending on their phenotypic β-lactam inactivation spectrums: “Narrow-Spectrum-β-lactamases” (NSBL), “Extended-Spectrum-β-lactamases” (ESBL), “Cephalosporinases” (AmpC), “NSBL with cefoxitin resistance” (AmpC-like), and “Carbapenemases” (CP) [2,3]. Amongst the Gram-negative bacteria, the first BLA enzyme was actually described in *Escherichia coli* [4], naturally present in the intestinal microbiota of humans and different animal species [5]. *E. coli* is also commonly involved in severe infections in both humans and animals [6]. Antibiotics, including β-lactams, are, therefore, widely used in human and veterinary medicine, contributing to the development of resistances [7,8]. Since the 1960s, *E. coli* has acquired several genes encoding many families of NSBL, ESBL, AmpC, and CP [2,3]. Moreover, these different BLA families can comprise several dozens of variants [9]. For instance, the most common ESBL enzymes, in humans as well as in animals, belong to the CTX-M family [10], which comprises more than 240 variants (available at: http://bldb.eu/BLDB.php?prot=A#CTX-M, last accessed 29 September 2021).

Since 2010, the World Health Organization (WHO), the World Organisation for Animal Health (OIE), and the Food and Agriculture Organization of the United Nations (FAO) have joined forces to fight antimicrobial resistance. They have proposed a list of “critically important antimicrobials for human medicine”, with restrictions on their use in veterinary medicine. In particular, the latest generation of cephalosporins, from third to fifth generation (3 GC to 5 GC), are categorised as “critically important and highest priority”, while the carbapenems, monobactams, aminopenicillins, and penicillins, both associated with β-lactams inhibitors, are “critically important” [11]. The resistance of bacteria in hospitals, including Gram-negative bacteria, to these last resort antibiotics is a major public health hazard worldwide [12]. A follow-up of these resistances in humans and animals was, therefore, applied in the European Union.

Even though there are significant differences between European countries, the highest resistance rate in humans is observed for the aminopenicillins (57.1%), followed by the 3 GC (15.1%)**,** while the resistance to carbapenems remains rare (0.3%) in *E. coli* [13]. In poultry, porcine, and bovine populations, the situation is similar for the aminopenicillins. Resistance to 3 GC is rare or at a very low level, except in Belgium and Lithuania. Fortunately, resistance to carbapenems has not been detected. In calves under 1 year of age, a high resistance rate to aminopenicillins (ampicillin), a lower level of resistance to 3 GC (cefotaxime), with a decrease between 2009 and 2017, and no resistance to carbapenems (meropenem) were reported [14]. The resistance to β-lactams of *E. coli* in the human population increased more slowly between 2015 and 2019 than between 2002 and 2012 [13], and the level of resistance to 3 GC (cefotaxime) in young calves decreased between 2009 and 2017 [14].

Different European countries have issued regulations for the use of antibiotics in food-producing animals. In Belgium, for instance, a Royal Decree was published in summer 2016: the use of carbapenems was prohibited and the use of 3 GC and 4 GC was strictly controlled [15]. As a possible consequence, a decrease in ESBL-producing pathogenic *E. coli* isolated from calves with diarrhoea and/or septicaemia was observed during the 2016–2017 calving season by the two Belgian routine diagnostic regional laboratories monitoring antimicrobial resistance amongst pathogenic enterobacteria from farm animals (cattle, small ruminants, pigs, and poultry): “Association Régionale de Santé et d’Identification Animales” (ARSIA) in Wallonia and “DierenGezondsheidZorg” (DGZ) in Flanders [16,17].

The purpose of this study was to compare the evolution of the resistance phenotypes to β-lactams of septicaemic and diarrheagenic *E. coli* isolated from young calves at ARSIA during seven calving seasons, from 2014–2015 to 2020–2021, with emphasis on the ESBL and AmpC phenotypes.

## 2. Material and Methods

### 2.1. E. coli Isolation

Pathogenic *E. coli* were isolated between November and February from 2014 to 2021 as part of the routine diagnostic procedure at ARSIA. They came (i) from faeces of diarrheic or intestinal contents of necropsied Belgian calves on the basis of a positive agglutination test (Biovac, Beaucouzé, France) for *fimbriae* F5 and/or F17a (enterotoxigenic *E. coli*) or for the CS31a surface antigen and/or on the basis of the production of an enterohaemolysin (Ehly) on washed sheep blood agar plates (90% are enteropathogenic and Shigatoxigenic *E. coli*) [18,19], or (ii) in pure culture from internal organs of necropsied calves suffering invasive infection.

A total of 3917 *E. coli* isolates from 3537 calves were analysed over seven calving seasons (Table 1).

### 2.2. Disk Diffusion Assay

The disk diffusion assay (DDA) was routinely performed at ARSIA on the pathogenic *E. coli* with 16 antibiotics, including 8 β-lactams: amoxicillin (AMX), amoxicillin + clavulanic acid (AMC), ceftiofur (XNL), cefquinome (CFQ), cefotaxime (CTX), cefotaxime + clavulanic acid (CTC), cefoxitin (FOX), and meropenem (MER) [16]. The whole procedure, including the analysis of the results, followed the European Committee on Antimicrobial Susceptibility Testing and “Comité de l’antibiogramme de la Société Française de Microbiologie” (EUCAST/CASFM) guidelines. The resistance profiles were classified in the five resistance categories as described earlier [2]: narrow-spectrum beta lactamases (NSBL: resistant to AMX and variable to AMC), extended spectrum beta lactamases (ESBL: resistant to AMX, XNL, CFQ and CTX), cephalosporinases (AmpC: resistant to AMX, AMC, XNL, CFQ, CTX, CTC and FOX), cephalosporinase-like (AmpC-like: resistant to AMX, AMC and FOX), and carbapenemases (CP: resistant to all eight beta-lactams).

### 2.3. Statistical Analysis

**Figure 1:**
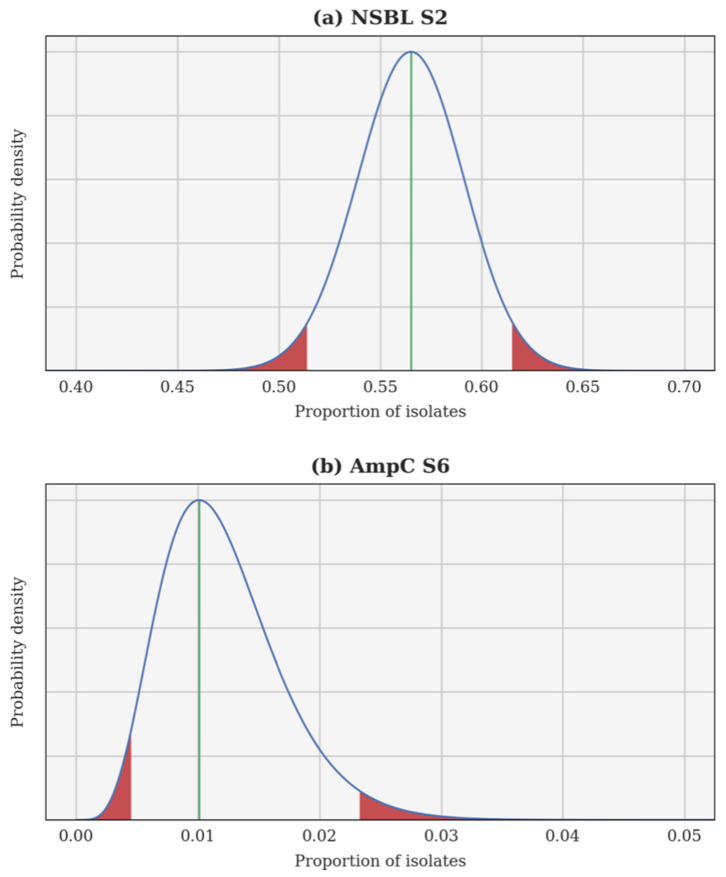
A posteriori probability density function of the theoretical proportion of resistant isolates p for the 2nd season narrow-spectrum β-lactamase (NSBL) (**a**) and for the 6th season cephalosporinase (AmpC) (**b**). In each case, the density is drawn using a continuous blue line, a vertical green line shows the maximum a posteriori, and the 95% confidence interval is delimited in red.

A Bayesian inversion is applied independently to each resistance category and each calving season. The proportion of resistant isolates p is treated as a random variable whose a priori probability density function (PDF) is uniform on the interval [0, 1]. Each individual test is a Bernoulli trial and, hence, the likelihood of observing a certain proportion of resistant isolates can be computed using a binomial distribution. Finally, the a posteriori PDF of p is obtained using Bayes’ theorem. This a posteriori PDF can be used to compute several estimates, such as the maximum a posteriori (most probable value of p) and the 95% confidence interval. In each case, the Bayesian inversion provides an a posteriori PDF for the theoretical proportion of resistant isolates p, as well as the associated 95% confidence interval. Two examples are illustrated in Figure 1: the 2nd season NSBL (2015–2016), for which the PDF of p is almost Gaussian and the 95% confidence interval is centred around the maximum a posteriori; and the 6th season AmpC (2019–2020), for which the PDF of p is skewed and the 95% confidence interval is not centred.

For each resistance category, the evolution of p over consecutive calving seasons is examined using a one-sided Fisher’s exact test. This evolution is considered significant if the *p*-value is lower than 0.05, in other words, if the probability of observing more extreme results is lower than 5% under the null hypothesis (when the evolution of p does not coincide with the observations). Furthermore, the a posteriori PDF can be used once again to compute the probability that the evolution of p coincides with the observations. This probability is a direct information on the evolution of p and constitutes a complementary diagnostic to confirm or infirm the statistical significance test (which is an indirect information on the evolution of p). The entire statistical analysis is performed using Python 3, using, in particular, SciPy’s implementation of Fisher’s exact test [20].

## 3. Results

### 3.1. Evolution of β-Lactam Resistance

**Figure 2:**
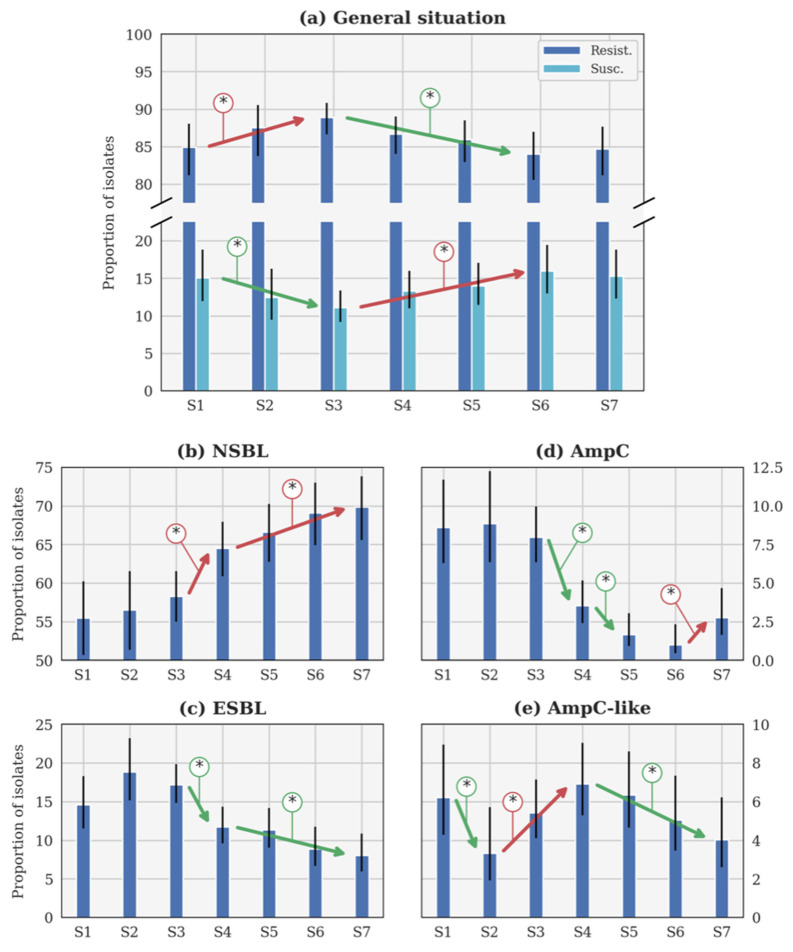
Evolution of the theoretical proportion of resistant isolates of the seasons (**a**) across all categories, as well as for (**b**) narrow-spectrum β-lactamase (NSBL), (**c**) extended-spectrum β-lactamase (ESBL), (**d**) cephalosporinase (AmpC), and (**e**) NSBL with nonsensitivity to cefoxitin (AmpC-like) categories. In each case, the blue histogram shows the maximum a posteriori and the black line shows the 95% confidence interval. The coloured lines with an asterisk show the statistically significant difference. S1: 2014–2015. S2: 2015–2016. S3: 2016–2017. S4: 2017–2018. S5: 2018–2019. S6: 2019–2020. S7: 2020–2021. Important note, scales were different for each phenotype for the clarity of the figures.

For each resistance category and each calving season, the number of resistant isolates, their origins, and the total number of tested isolates are reported in Table 2. The results of Fisher’s exact test (the *p*-value) and the Bayesian inversion (the probability) for the evolution of each resistance category are reported in Table 3. The evolution of p over the seasons is illustrated for each category in Figure 2.

#### 3.1.1. General Evolution of β-Lactam Resistance

The general β-lactam resistance increased from S1 to S3, but decreased between S3 and S6 (Figure 2a). This evolution is statistically significant, contrary to the small increase between S6 and S7. Conversely, the susceptible proportions statistically decreased between S1 and S3, before increasing until S6 (Figure 2a). The evolution between S1 and S7 is not significant according to the *p*-value (Table 3). Since no CP was detected, this profile will not be further described in the following sections.

#### 3.1.2. Evolution of the NSBL Profile

The only significant evolution of p between consecutive seasons (Figure 2b) was the increase between S3 and S4 (respectively, 2016–2017 and 2017–2018). The increase was slower between each season from S4 (2017–2018) to S7 (2020–2021), but still significant. It is remarkable that the rate of NSBL resistance significantly increased from S1 to S7 (Table 3).

#### 3.1.3. Evolution of the ESBL Profile

In Figure 2c, p was higher during S2 (2015–2016) than during S1 and S3 (respectively, 2014–2015 and 2016–2017). Such evolutions were highly probable (probability higher than 0.95 in both cases) but not significant according to the *p*-value (*p*-value higher than 0.05 in both cases). Beyond this specific point, the only significant evolution of p between consecutive seasons was the decrease between S3 (2016–2017) and S4 (2017–2018). The decrease was slower between S4 and S7, but still significant between these two seasons. The decrease between S1 and S7 is significant according to the *p*-value (Table 3).

#### 3.1.4. Evolution of the AmpC Profile

Three significant evolutions of p between consecutive seasons were observed in Figure 2d. Two distinct decreases were observed between S3 (2016–2017) and S4 (2017–2018), and S4 and S5 (2018–2019), while an increase between S6 (2019–2020) and S7 (2020–2021) was reported. However, a significant decrease is observed between S1 and S7, according to the *p*-value (Table 3).

#### 3.1.5. Evolution of the AmpC-like Profile

The only significant evolution of p between consecutive seasons (Figure 2e) was the decrease between S1 and S2 (respectively, 2014–2015 and 2015–2016). Later, a significant increase was observed between S2 and S4 (2015–2016 and 2017–2018), followed by a slow decrease that was significant between S4 and S7 (2017–2018 and 2020–2021).

### 3.2. Comparison of β-Lactam Resistance Profiles between the Escherichia coli Isolates by Origin

The tested *E. coli* were isolated from the faeces of diarrheic calves or from the intestinal content or internal organs of necropsied calves. Table 4 presents the results of the comparison between *E. coli* from each site of origin for each resistance profile and each calving season. This analysis highlighted differences in the resistance profile depending of the origin of *E. coli*. Indeed, faecal *E. coli* are usually characterised by NSBL and ESBL profiles, whereas *E. coli* isolated from necropsied calves are usually characterised by AmpC and AmpC-like profiles.

### 3.3. Comparison of β-Lactam Resistance Profiles between the Escherichia coli Virulotypes

The *E. coli* tested belonged to different virulotypes (F5+, F17+, CS31a+, or Ehly+). Table 5 presents the resistance profile repartition of these different *E. coli* for all calving seasons. The analysis of each calving season considered separately was not pertinent, due to the very small number of isolates in some categories. Therefore, no association between virulotypes and resistance profile was statistically significant.

## 4. Discussion

The evolution observed in the resistance rates is consistent with those presented in the European Food Safety Authority (EFSA) report, even if the origin of the *E. coli* differs. In this study, the *E. coli* were isolated from diarrheic or septicaemic calves, while, in the EFSA report, they were collected from slaughterhouse specimens [14]. No association between resistance and virulence could be determined from the phenotypes identified. This observation should be confirmed in future studies, with analysis of the genetic basis of these two aspects.

Three effects of the Royal Decree of 2016 [15] were observed.

Firstly, because of the Royal Decree of 2016 [15], more than twofold more susceptibility tests were performed during S3 than in S2 (Table 2).

The second effect of the Royal Decree [15] was a decrease in resistance to cephalosporins (ESBL, AmpC, and AmpC-like resistance profiles), with different rates over the seasons. Indeed, the ESBL profile decreased slowly (around 16% at S3 to 7.5% at S7), while the AmpC profile followed the same tendency (8% at S3 to 1% at S6), except during the last calving season, when an increase was observed. These observations may be linked to the variation in the use of 3 GC and 4 GC in livestock, even though there is a lack of data for the use of 3 GC and 4 GC for systemic use in cattle [21]. The decrease in the AmpC-like profile starts at S4 instead of S3. This may be explained because this profile harbours a resistance to second generation cephalosporins (2 GC) and not to 3 GC/4 GC. Restriction on their use is, therefore, not the main cause of this change.

The third effect of the Royal decree [15] was an increase in NSBL, which may be a consequence of the change in the first-line treatment at the farm level. The regulation on the use of 3 GC and 4 GC resulted in a decrease in their use and an increasing use of the penicillins in first-line treatment, as well as for some 1 GC, even if there was a global decrease in the quantity of antibiotics used [22]. These observations highlight the importance of a monitoring of each resistance profile and of the extension of this monitoring to all animal species. This will be possible in the future thanks to the recently created European Antimicrobial Resistance Surveillance—Veterinary (EARS-Vet) network, which will follow and study the antibiotic resistances in bacteria from sick animals [22], similarly to the EARS-net for human medicine [13].

To explain the increase in some phenotypes, as well as the slow decrease in others, the most likely hypothesis is a coselection effect. Indeed, the use of another family of antibiotics may also select a β-lactam resistance. For instance, to treat severe infection due to Gram-negative bacteria in humans, aminoglycosides are used frequently in combination with β-lactam and a co-occurrence of resistance is observed [23]. The localisation of resistance-encoding genes is also an important point, especially for BLA enzymes that are, most of the time, encoded by plasmid-located *bla* genes. These plasmids may harbour different resistance genes in addition to *bla* genes, such as those encoding enzymes hydrolysing aminoglycosides or for resistance to quinolones and sulphonamides [24]. That may explain that the use of other antibiotics, in the context of regulation on 3 GC and 4 GC utilisation [15], such as sulphonamides or aminoglycosides [23], may maintain the resistance to β-lactam in the bovine population [24].

Differences observed between the two origins of *E. coli*, faeces from diarrheic calves or intestinal content or organs from necropsied calves, may be linked to the hypothesis that, most of the time, the necropsied calves were treated with antibiotics before death. Especially since, due to their economic value as meat calves, preventive treatment is often used when there are problems on the farm. The higher frequency of AmpC and AmpC-like resistance profiles may, therefore, be linked, under the previous hypothesis, to the preventive use of some β-lactam or other antibiotics with associated resistance of the type previously discussed. In addition, the almost systematic use of antibiotics when performing caesarean sections without a defined protocol [25] could also influence the resistance observed in beef calves. In dairy production, the intra-mammary use of β-lactams is also very frequent during mastitis or when drying off [26]. They are, moreover, the most widely used antibiotics in Belgium [21] and residues of these antibiotics can be found in milk, which, if given, even pasteurised, to calves will influence the resistance rate of *E. coli* [27]. Further analyses will be necessary to improve the understanding of this link between resistance and antibiotic utilisation.

The use of β-lactam antibiotics is widely spread and penicillins are the most used β-lactams, either in human [28] or in veterinary medicine [29]. This study confirms that compulsory regulation leading to a decrease in the use of 3 GC/4 GC is associated with a decrease in the associated resistances. Although, beyond the scope of this study, this observation raises the question of whether this type of regulation should also be applied to human medicine at the national level in the One Health context [30].

To conclude, the major finding of this study was the positive effect of the Royal Decree on the level of resistance to 3 GC and 4 GC, with an inversion of the curve. However, some resistance profiles presented an increase. To understand the consequences of the Royal Decree more precisely in the long term, it will be necessary to continue this surveillance in the future.

## Figures and Tables

**Table 1:** Number of *Escherichia coli* isolates analysed per calving season and the associated number of calves.

Calving Seasons	Number of *E. coli* Isolates	Number of Calves
S1: 2014–2015	418	384
S2: 2015–2016	361	334
S3: 2016–2017	866	740
S4: 2017–2018	707	646
S5: 2018–2019	599	545
S6: 2019–2020	495	464
S7: 2020–2021	471	424
Total overall calving seasons	3917	3537

**Table 2:** Number and proportion of *Escherichia coli* isolates tested by disk diffusion assay for each calving season (from 2014–2015 to 2020–2021).

Calving Season and Origin of *Escherichia coli*	Resistance Profiles (%)					Total
	Susceptible	NSBL	ESBL	AmpC	AmpC-like	
S1	63	232	61	36	26	418
	(15.1)	(55.5)	(14.6)	(8.6)	(6.2)	(100.0)
Faeces	28	147	28	12	13	228
	(6.7)	(35.2)	(6.7)	(2.9)	(3.1)	(54.5)
Intestinal content + organs	35	85	33	24	13	190
	(8.4)	(20.3)	(7.9)	(5.7)	(3.1)	(45.5)
S2	45	204	68	32	12	361
	(12.5)	(56.5)	(18.8)	(8.9)	(3.3)	(100.0)
Faeces	20	114	33	11	6	184
	(5.5)	(31.6)	(9.1)	(3.0)	(1.7)	(51.0)
Intestinal content + organs	25	90	35	21	6	177
	(6.9)	(24.9)	(9.7)	(5.8)	(1.7)	(49.0)
S3	96	505	149	69	47	866
	(11.1)	(58.3)	(17.2)	(8.0)	(5.4)	(100.0)
Faeces	55	301	85	31	20	492
	(6.4)	(34.8)	(9.8)	(3.6)	(2.3)	(56.8)
Intestinal content + organs	41	204	64	38	27	374
	(4.7)	(23.6)	(7.4)	(4.4)	(3.1)	(43.2)
S4	94	456	83	25	49	707
	(13.3)	(64.5)	(11.7)	(3.5)	(6.9)	(100.0)
Faeces	51	304	51	11	34	451
	(7.2)	(43.0)	(7.2)	(1.6)	(4.8)	(63.8)
Intestinal content + organs	43	152	32	14	15	256
	(6.1)	(21.5)	(4.5)	(2.0)	(2.1)	(36.2)
S5	84	399	68	10	38	599
	(14.0)	(66.6)	(11.4)	(1.7)	(6.3)	(100.0)
Faeces	57	309	42	5	29	442
	(9.5)	(51.6)	(7.0)	(0.8)	(4.8)	(73.8)
Intestinal content + organs	27	90	26	5	9	157
	(4.5)	(15.0)	(4.3)	(0.8)	(1.5)	(26.2)
S6	79	342	44	5	25	495
	(16.0)	(69.1)	(8.9)	(1.0)	(5.1)	(100.0)
Faeces	48	251	31	3	18	351
	(9.7)	(50.7)	(6.3)	(0.6)	(3.6)	(70.9)
Intestinal content + organs	31	91	13	2	7	144
	(6.3)	(18.4)	(2.6)	(0.4)	(1.4)	(29.1)
S7	72	329	38	13	19	471
	(15.3)	(69.9)	(8.1)	(2.8)	(4.0)	(100.0)
Faeces	35	220	28	7	16	306
	(7.4)	(46.7)	(5.9)	(1.5)	(3.4)	(65.0)
Intestinal content + organs	37	109	10	6	3	165
	(7.9)	(23.1)	(2.1)	(1.3)	(0.6)	(35.0)
Total of 7 seasons	533	2467	511	190	216	3917
	(13.6)	(63.0)	(13.0)	(4.9)	(5.5)	(100.0)

NSBL: narrow-spectrum β-lactamase. ESBL: extended-spectrum β-lactamase. AmpC: cephalosporinase. AmpC-like: NSBL with nonsensitivity to cefoxitin. S1: 2014–2015. S2: 2015–2016. S3: 2016–2017. S4: 2017–2018. S5: 2018–2019. S6: 2019–2020. S7: 2020–2021.

**Table 3:** Results of Fisher’s exact test (*p*-value) for each resistance profile over the seven calving seasons.

Sensitive		NSBL		AmpC-like		AmpC		ESBL		Overall Resistance	
Season Comparison	*p*-Value	Season Comparison	*p*-Value	Season Comparison	*p*-Value	Season Comparison	*p*-Value	Season Comparison	*p*-Value	Season Comparison	*p*-Value
S1 > S2	0.17220	S1 < S2	0.41691	S1 > S2	0.04287	S1 < S2	0.50021	S1 < S2	0.06798	S1 < S2	0.17220
S2 > S3	0.27458	S2 < S3	0.30130	S2 < S3	0.07414	S2 > S3	0.33818	S2 > S3	0.27266	S2 < S3	0.27458
S3 < S4	0.10395	S3 < S4	0.00708	S3 < S4	0.12870	S3 > S4	0.00013	S3 > S4	0.00140	S3 > S4	0.10395
S4 < S5	0.38117	S4 < S5	0.22915	S4 > S5	0.37838	S4 > S5	0.02655	S4 > S5	0.44848	S4 > S5	0.38117
S5 < S6	0.20884	S5 < S6	0.20953	S5 > S6	0.21715	S5 > S6	0.25295	S5 > S6	0.10761	S5 > S6	0.20884
S6 > S7	0.42125	S6 < S7	0.42607	S6 > S7	0.27372	S6 < S7	0.03709	S6 > S7	0.36649	S6 < S7	0.42125
Supplementary comparison tests											
S1 < S7	0.50232	S1 < S7	0.00001	S1 > S7	0.09170	S1 > S7	0.00010	S1 > S7	0.00143	S1 > S7	0.50232
S1 > S3	0.02725	S1 < S3	0.18548	S2 < S4	0.00968	NN	NN	S1 < S3	0.13417	S1 < S3	0.02725
S3 < S6	0.00664	S4 < S7	0.03217	S4 > S7	0.02329	NN	NN	S4 > S7	0.02538	S3 > S6	0.00664

NSBL: narrow-spectrum β-lactamase. AmpC-like: NSBL with nonsensitivity to cefoxitin. AmpC: cephalosporinase. ESBL: extended-spectrum β-lactamase. S1: 2014–2015. S2: 2015–2016. S3: 2016–2017. S4: 2017–2018. S5: 2018–2019. S6: 2019–2020. S7: 2020–2021. NN: supplementary test not necessary in this case. Significant *p*-values are underlined.

**Table 4:** Results of Fisher’s exact test (*p*-value) for each resistance profile of *Escherichia coli*, depending on their origin, over seven calving season (from 2014–2015 to 2020–2021).

*Escherichia coli* from Diarrheic vs. Necropsied Calves	Resistance Profiles					Global Resistance
	Susceptible	NSBL	ESBL	AmpC	AmpC-like	
S1	0.09914	0.00007	0.16452	0.00856	0.68683	0.09914
S2	0.42590	0.03446	0.68770	0.06327	1.00000	0.42590
S3	1.00000	0.05172	1.00000	0.04268	0.04899	1.00000
S4	0.04960	0.03377	0.62907	0.05420	0.44391	0.04960
S5	0.18336	0.00569	0.01947	0.13785	0.84952	0.18336
S6	0.04195	0.08637	1.00000	0.63112	1.00000	0.04195
S7	0.00200	0.20700	0.28907	0.39208	0.08722	0.00200

S1: 2014–2015. S2: 2015–2016. S3: 2016–2017. S4: 2017–2018. S5: 2018–2019. S6: 2019–2020. S7: 2020–2021. NSBL: narrow-spectrum β-lactamase. ESBL: extended-spectrum β-lactamase. AmpC: cephalosporinase. AmpC-like: NSBL with nonsusceptibility to cefoxitin. Significant *p*-values are underlined.

**Table 5:** Repartition of the various *Escherichia coli* virulotypes over the resistance profiles, with all seven calving seasons considered together.

*Escherichia coli* Virulotype	Resistance Profiles in %					Total
	Susceptible	NSBL	ESBL	AmpC	AmpC-like	
F5	0.2	5.8	0.3	0.1	−	6.3
F17a	3.0	12.5	2.3	1.8	1.7	21.4
CS31A	4.1	34.5	7.9	1.5	2.8	50.9
Ehly+	2.3	1.5	0.2	0.1	0.1	4.1
ND	4.0	8.6	2.4	1.4	0.9	17.3
Total	13.6	63.0	13.1	4.9	5.5	100.0

F5: *fimbriae* F5 expressed by *Escherichia coli*. F17a: *fimbriae* F17a expressed by *Escherichia coli*. CS31a: surface antigen CS31a expressed by *Escherichia coli*. Ehly+: *Escherichia coli* producing an enterohaemolysin. ND: *Escherichia coli* with virulence factors not determined. NSBL: narrow-spectrum β-lactamase. AmpC-like: NSBL with nonsusceptibility to cefoxitin. AmpC: cephalosporinase. ESBL: extended-spectrum β-lactamase. −: No isolates in this category were found.
